# Supplementary material for: Optimal predictive probability designs for randomized biomarker-guided oncology trials
Source: Front Oncol. 2022 Dec 6;12:955056. doi: 10.3389/fonc.2022.955056 (PMC9763994; doi:10.3389/fonc.2022.955056)
Supplement: Supplementary file 1 [file Table_1.pdf]

	Pooled	Stratified	Enrichment	
			Stage 1	Stage 2
Type I error	0.064 (0.009)	0.036 (0.005)	0.103 (0.017)	0.089 (0.006)
Power	0.820 (0.012)	0.813 (0.009)	0.754 (0.032)	0.889 (0.010)
Avg N Null	108.10 (3.66)	155.6 (4.7)	107.24 (4.35)	
Avg N Alt	156.76 (2.44)	222.5 (4.2)	224.7 (5.0)	

Supplemental Table 1. Mean (standard deviation) of operating characteristics across a range of six different Beta priors.

	Pooled		Stratified		Enrichment			
					Stage 1		Stage 2	
	Type I error	Power	Type I error	Power	Type I error	Power	Type I error	Power
IC0	0.07	0.81	0.08	0.82	--	--	--	--
IC1	0.08	0.82	0.09	0.80	--	--	--	--
IC2/3	0.07	0.82	0.08	0.81	--	--	--	--
Overall	--	--	--	--	0.06	0.67	0.09	0.83

Supplemental Table 2. Type I error and power for each biomarker-specific subgroup under the pooled control arm and stratified control arm designs, and the overall type I error and power for the enrichment design. For the homogeneous response setting.

	Pooled		Stratified		Enrichment	
	Avg N Null	Avg N Alt	Avg N Null	Avg N Alt	Avg N Null	Avg N Alt
Control	32.1	49.2	73.2	136.2	29.6	79.8
IC0	23.6	45.0	24.2	45.6	--	--
IC1	24.6	45.0	24.6	45.3	--	--
IC2/3	25.0	45.2	24.4	45.3	--	--
Total Atezolizumab	73.2	135.2	73.2	136.2	61.3	159.0
Total Enrolled	105.3	184.4	146.4	136.2	90.9	239.0

Supplemental Table 3. Average sample size under the null (“Avg N Null”) and average sample size under the alternative (“Avg N Alt”) by design and treatment subgroup. For the homogeneous response setting.
